# Risky sexual practice and associated factors among street children of Wonago town, Gedeo zone, Southern Ethiopia

**DOI:** 10.3389/fpubh.2023.1089499

**Published:** 2023-04-05

**Authors:** Getachew Assefa Zenebe, Wagaye Alemu, Temesgen Muche, Berhanu Gidisa Debela

**Affiliations:** School of Public Health, College of Medicine and Health Sciences, Dilla University, Dilla, Ethiopia

**Keywords:** risky sexual practice, street children, substance abuse, Gedeo zone, Ethiopia

## Abstract

**Introduction:**

A “risky sexual practice” is any sexual act that might put an individual’s social, physical, and psychological health at risk and increase the likelihood of adverse sexual and reproductive health consequences. Street children have risky sexual practices, which place them at all kinds of health risks. Therefore, the aim of this study is to assess risky sexual practices and associated factors among street children in Wonago Town, southern Ethiopia.

**Methods:**

A community-based cross-sectional study design was employed. About 214 street children, aged 10–18, residing in Wonago Town from September 1–30, 2021, participated in the study. Data was gathered from all of the street children in Wonago town. A pre-tested and structured interviewer-administered questionnaire was used to collect data. Epi data software was used to enter the collected data, which was then exported to SPSS for analysis. A logistic regression model was applied to identify the associated factors. A *p* value <0.05 was used to declare the significant variables.

**Results:**

A total of 214 street children were involved in the study. Overall, the magnitude of risky sexual practices was 43.46 percent (95% CI: 38.3–48.62). About 52.7% of participants had ever had sexual intercourse in the last year, and of them, around 43.5% had more than one sexual partner. Among sexually active street children, 40.9% never used a condom during sexual intercourse. Age (Adjusted Odd Ratio (AOR): 1.42, 95%CI: 1.03–2.37), educational status (AOR: 5.73, 95%CI: 1.49–10.51), substance use (AOR: 1.24, 95%CI: 1.03–2.07), duration on the street (AOR: 2.14, 95%CI: 1.03–4.12), and daily income (AOR: 0.68, 95%CI: 0.32–0.98) were found to be significantly associated with risky sexual practices.

**Conclusion:**

Risky sexual practices among street children were more prevalent in Wonago Town, Southern Ethiopia. Children’s age, educational status, daily income, duration on the street, and status of substance use were the factors that contributed to risky sexual practices. As a result, conducting information and education campaigns, developing income-generating activities for children, increasing children’s school enrollment and attendance, improving access to sexual and reproductive health services, and providing capacity-building training for health care providers may all help to reduce risky sexual practices.

## 1. Introduction

Street children are what the United Nations Children’s Fund (UNICEF) refers to as “children in challenging situations,” a minority group that has for far too long been underrepresented in health studies. Street children have been categorized into two overlapping categories: “of-the-street” children, who have no contact with family and infrequently go home, and “on-the-street” children, who frequently spend the night at home but spend the day on the streets (1, 2).

The United Nations estimates there are up to 150 million street children in the world (3), but according to a recent estimate by Ethiopia’s Ministry of Labour and Social Affairs, Addis Ababa had about 24,000 homeless residents in 2018—roughly 10,500 of whom were children, and about 13,500 homeless adults (4). Similarly, evidence indicates that over four million children in Ethiopia are expected to live under challenging conditions (5). They are at high risk of being physically and sexually exploited, as well as engaging in risky sexual practices (6).

Risky sexual practice is defined as any sexual act that might put an individual’s social, physical, and psychological health at risk (7). Many authors defined risky sexual practice as having multiple sexual partners, early initiation of sex, inconsistent condom use, sex with commercial sex workers, and unprotected sex with a same-sex partner, especially when it involves male partners (8–11). Personality and physiology play a significant role in determining a person’s sexual practice (12). It is typically associated with other risky behaviors such as substance abuse, poor academic performance, and violent behavior (7).

Risky sexual practice can have harmful sexual and reproductive health consequences, like unwanted pregnancy, unsafe abortion, acquired immunodeficiency syndrome (AIDS) or human immunodeficiency virus (HIV), sexually transmitted diseases (STDs), and being in a sexual relationship before being mature enough to know what constitutes a healthy relationship. People may engage in risky practices because they may not understand the concerns about HIV/AIDS and STDs, like signs and symptoms, mode of transmission, and preventive measures. Drugs and alcohol impair judgment and make unsafe sexual behavior more likely (13, 14).

Teenagers and young adults are more likely than adults to engage in risky sexual practices (13), which may increase the sexual and reproductive health risks of street children in general and female street children in particular (15). Street children live a transitory lifestyle and are vulnerable to inadequate nutrition, physical injuries, substance use, and health problems, including sexual and reproductive health problems (16). They are exposed to street subcultures such as smoking, drugs, alcohol, substance abuse, gambling, sexual activities, and selling sex for survival (17). The circumstances in which they live and work increase their vulnerability to sexual exploitation and abuse and put them at a higher risk of sexual and reproductive health outcomes (18).

Evidence showed that about 61.9% of the street children in Brazil (19), 55% in Kenya (20), 61.7% (21) and 62.4% (22) in Gondar, and 53.9% (23) and 31.6% (15) in Southern Ethiopia were practicing risky sexual activity.

Street children do not have the kinds of connections to important childhood institutions like family, education, and health that society deems suitable. They are susceptible to substance abuse due to the nature of their lifestyles and ongoing exposure to harsh environments, which endangers their mental, physical, social, and spiritual wellbeing (16, 19). Despite these alarming realities, street children rarely have a voice in the sexual and reproductive health discourse. Governmental and nongovernmental organizations’ intervention programs are not based on the realities of street children. This is because these organizations work through existing societal structures such as hospitals, schools, local communities, and facilities from which street children are disconnected (24).

Despite the fact that risky sexual practices are common among street children, evidence assessing the factors that lead to these practices is scarce and, when available, incomplete. As a result, more research is required to comprehend and address the issue of risky sexual practices. Hence, undertaking a study in this area is believed to provide information on risky sexual practices and their determinants, and relevant information was generated that could help organizations design an appropriate sexual and reproductive health program and improve future services for this disadvantaged segment of the population to minimize risky sexual practices and their consequences among street children. Therefore, the aim of this study was to assess risky sexual practice and associated factors among street children in Wonago Town, Gedeo Zone, Southern Ethiopia, in 2021.

## 2. Materials and methods

### 2.1. Study setting

This study was conducted in Wonago town, Gedeo zone, Southern Nations, Nationalities, and Peoples’ Region (SNNPR). It is located 105 km from Hawassa (the capital city of the South Region) and 15 km from Dilla (the capital town of the Gedeo zone) on the main road from Dilla to Moyale. The town is surrounded on the north by Dilla Zuria, on the south by Yirgaceffe, on the east by Bule Woreda, and on the west by Abaya Zuria Woreda of the west Guji Zone of Oromia Region. Based on the 2007 national census conducted in Ethiopia, the woreda has a total population of 116,921, of whom 58,150 are men and 58,771 women; 8,471 or 7.25% of its population are urban dwellers. The majority (76.11%) of the inhabitants were Protestant believers (25). Currently, the town has one health center, seven health posts, and three private clinics. The health center provides HIV/AIDS treatment and care services for the woreda and neighboring communities. The Woreda annual report showed that about 7, 32, and 6 new HIV positive clients were registered in the facility in 2020, 2021, and 2022, respectively. Among them, about five HIV-positive cases were from voluntarily tested street children (26).

### 2.2. Study design and study period

A community-based cross-sectional study design was employed among street children residing in Wonago town from September 1–30, 2021.

### 2.3. Population

All the street children who live in Wonago town were the source population, whereas those children available during the data collection period were the study population. Each street child was the study unit.

### 2.4. Inclusion and exclusion criteria

All street children in Wonago town between the ages of 10 and 18 were included in the study, whereas street children who had recently joined the street (3 months) were excluded.

### 2.5. Sample size determination, sampling techniques, and procedures

To develop the sampling frame, first a census (a short-form census) was conducted to determine the number of street children in the age group of 10–18 in Wonago town in collaboration with the office of labor and social affairs and women, children, and youth affairs. The census report showed that the total number of street children in the town was 235. Since it was feasible to collect data from all respondents, there was no need to calculate a sample size, and data was collected from all street children.

### 2.6. Study variables

Risky sexual practice was the dependent variable, whereas sex, age, educational status, daily average income, duration on the street, with whom one is living, and place of residence before joining street life were the independent variables.

### 2.7. Operational definitions

#### Risky sexual practice

Street children who had sex with a non-regular sexual partner or exchanged sex for money, or had more than one sexual partner, or had been raped in the last twelve months, or did not use condoms or used them inconsistently in the last three months. Those respondents who had one or more of these were considered to have risky sexual practices (15).

#### Street children

They are children less than 18 years old, living on and off the street. They are children in difficult circumstances who struggle to survive in the city (1, 2).

#### Drug/Substance

Any substance that, when taken into the living organism, may modify one or more of its functions. In this study, the concept of drug covers substances such as alcoholic drinks, tobacco, chat, hashish, and benzene (15).

### 2.8. Data collection tool, procedure, and data quality management

The data was collected using a structured interviewer-administered questionnaire. It was adapted and modified after reviewing different related literature as appropriate to address the study objectives (15, 17). The content includes risky sexual practice questions as an outcome variable, socio-demographic and economic variables, individual risk factors (alcohol intake and substance abuse), and health service-related factors as independent variables. The questionnaire was originally developed in English and then translated into the local Amharic, Gedeuffa, and Oromiffa languages and back to English. Before data collection, a day of training was given to the four data collectors (reproductive health masters students). The questionnaire was pre-tested on 5% (12 participants) of our sample in Chichu woreda, which has a similar population, with the possibility of modifying the questionnaire. We checked or labeled each participant’s fingernails with permanent marker in order to reduce participant interview repetition because street children move from one location to another. The data is manually reviewed and checked for completeness, accuracy, and clarity by the authors each day following data collection. They also give the appropriate feedback to the data collectors.

### 2.9. Data processing and analysis

After data collection, the response was coded and entered using Epi Data version 4.2 software and then exported to the Statistical Package for Social Science (SPSS) version 23 for data analysis. Descriptive statistics were presented in tables and figures using percentages, means, and frequency distributions. To select the appropriate model for this study, those multiple questions for the outcome variable were computed to merge into a single outcome variable. During computing in the SPSS software, participants who said yes at least for a single question were considered risky (“yes”) for the outcome category but not risky (“no”) otherwise. Then, binary logistic regression was fitted to determine the association of independent variables with risky sexual practice after the assumption was fulfilled. Variables with *p* values of 0.2 were included in multivariable analysis. In the final model, adjusted odds ratios (AOR) with 95% CI and a value of *p* <0.05 were used to declare the significant variables.

## 3. Results

### 3.1. Socio-demographic and economic characteristics of the respondents

A total of 214 street children participated in the study, with a 91.06% response rate. Of them, the majority (94.9%) of the participants were males. The age range of the respondents was 10–18 years. Around half (49.1%) of participants were born in rural areas, and the remaining were in urban areas. Regarding the educational status, about 52.8% of participants attended elementary school up to grade six. The main reasons for joining street life were looking for a job (37.4%) and poverty (35.5%). About 3/4 of the participants have lived on the street for more than a year. More than half (58.4%) of participants live with their peers on the street (Table 1).

**Table 1:** Socio-demographic and economic characteristics of street children in Wonago town, Southern Ethiopia, 2021.

Variables response		Frequency	Percent
Sex	Male	203	94.9%
	Female	11	5.1%
Age	10–15	79	36.9%
	16–18	135	63.1%
Place of birth	Urban	109	50.9%
	Rural	105	49.1%
Educational level	No formal education	87	40.7%
	Grade 1–6	113	52.8%
	Grade 7–12	14	6.5%
Religion	Orthodox	67	31.3%
	Protestant	109	50.9%
	Muslim	16	7.5%
	Other	22	10.3%
Types of street children	On the street	143	50.35%
	Off the street	71	29.57%
Street children’s reason to live on the street	Looking for job	80	37.4%
	Poverty	76	35.5%
	Family disharmony	15	7.0%
	Death of parent	20	9.3%
	Peer pressure	6	2.8%
	Other	17	7.9%
Duration on the street	< 1 year	53	24.8%
	>1 year	161	75.2%
Currently live with whom	Peer	125	58.4%
	Alone	56	26.2%
	Boy/girl friend	7	3.3%
	Parent	22	10.3%
	Others (relatives, elders)	4	1.9%
Source of income	Carrying items	62	44%
	Begging	27	19%
	Shoeshine	39	27%
	Care washing	12	9%
	Others	14	1%
Average daily income (in Ethiopian Birr (ETB))/USD	<20 ETB (0.43 USD)	141	65.9%
	≥21ETB (≥0.45USD)	73	34.1%
Do you ever get support from any organization?	1.Yes, currently	10	4.63%
	Yes, but not now	32	15%
	Not at all	172	80.37

Regarding their income, the major sources of income for the children were carrying items (44%), and the least one was car washing (9%) and commercial sex work (1%). Around 65.9% of respondents had an average daily income of less than 20 Ethiopian birr (0.45 USD). Concerning support, only 4.6% of respondents received support from a locally available volunteer organization; 15% received assistance from a non-governmental organization that was in place to assist street children in any psychosocial aspect; and approximately 80.4% received no assistance from any organization (Table 1).

### 3.2. Individual risk factors

#### 3.2.1. Alcohol intake and substance abuse

**Figure 1:**
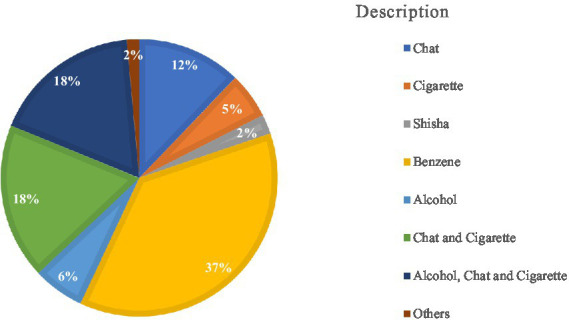
The types of substances consumed by the substance user of street children in Wonago town, September 2021 (*N* = 132).

More than half (61.7%) of respondents used substances in the last year. Among users, benzene (37%), chat and cigarettes (18%), and a combination of alcohol, chat, and cigarettes (18%) are the most commonly used substances (Figure 1). The common reasons for substance abuse were to avoid depression (47.6%), peer influence (22.0%), hunger (17.3%), and frustration (10.5%). Out of 132 (61.7%) substance-using street children, about 39.45% of respondents were initiated into risky sexual practices due to substance use.

### 3.3. Health service-related factors

**Figure 2:**
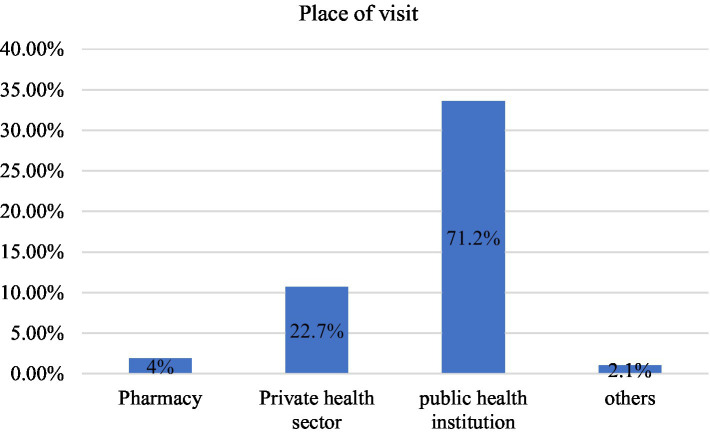
Type of health institution frequently visited by street children for SRH services in Wonago town, September 2021 (*N* = 101).

Nearly half, 113 (52.8%), of the respondents did not visit a health institution for sexual and reproductive health services (SRS) in the last year. Among visitors, the major reason for visiting was sexually transmitted infections (26.7%), and the major barrier for not visiting health institutions was the attitude of health workers (24.6%) (Table 2). Around 3/4th (71.2%) of the respondents visited public health institutions when they needed services (Figure 2).

**Table 2:** Common barriers of street children’s for not visiting health institutions for SRH services in Wonago town, September 2021 (*N* = 113).

Barrier for not visiting SRH services	Frequency	Percent
Attitude/Poor handling by health worker	28	24.6%
Lack of money	69	61.4%
Too far	4	3.5%
Fail to keep privacy and confidentiality	8	7%
Others	4	3.5%

### 3.4. Risky sexual practice

**Figure 3:**
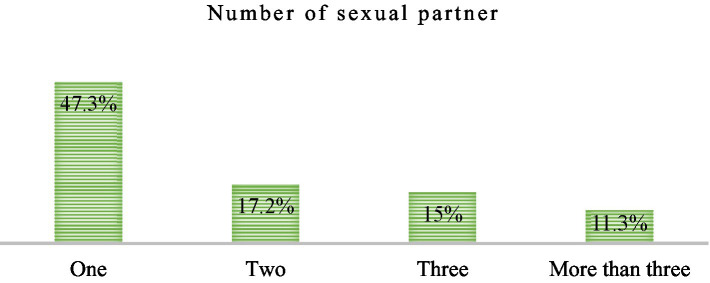
Sexually active street children’s sexual partner in Wonago town, September 2021 (*N* = 93).

Overall, the magnitude of risky sexual practices was 43.46% (95% CI: 38.3–48.62). About 52.7% of participants had ever had sexual intercourse in the last year, and of them, around 43.5% had more than one sexual partner (Figure 3). About 30.4% of them started sex in the age group of 10–14 years. Falling in love (34.4%) and exchanging something for money (16.1%) are the two major factors in having sex (Table 3).

**Table 3:** Sexually active street children’s reason to had sex in Wonago town, September 2021 (*N* = 93).

Reason to had sex	Frequency	Percent
Exchange for money	15	16.1%
Fall in love	32	34.4%
Substance influence	21	22.5%
Marriage	2	2.2%
Peer pressure	12	12.9%
Personal desire	9	9.7%
Rape	2	2.2%

Among 93 sexually active street children, only 59.1% used condoms; the rest, 40.9 percent (38), never used condoms during sexual intercourse, and the majority of them got the service from health centers, hospitals, and pharmacies (63.7%) (Table 4).

**Table 4:** Sexually active street children’s condom utilization habit in Wonago town, September 2021 (*N* = 55).

Variables		Frequency	Percent
Places where sexually active street children get condom	Health center/hospital	20	36.4%
	Pharmacy	15	27.3%
	Shop	8	14.5%
	Other (e.g., friends, “Suk bederite”)	12	21.8%
Frequency of condom use	Frequently/always	21	38.2%
	Inconsistently/sometimes	34	61.8%
*Reason for not using condom*			
Condom decrease sexual pleasure		3	7.9%
Lack of access to condom		18	47.3%
My partner did not want to use		2	5.3%
The situation is accidental		13	34.2%
Do not know how to use		2	5.3%

### 3.5. Factor associated with risky sexual practice

In bivariate analysis, age, educational status, daily average income, with whom one is living, substance use, place of residence before joining street life, types of street children, duration on the street, and support from any organization were fitted into multivariable analysis. In the final multivariable analysis, age, educational status, substance use, duration on the street, and daily income were found to be significantly associated with risky sexual practice.

Accordingly, being in the age group of 16–18 made one 42% more likely to engage in risky sexual activity as compared to those in the age group of 10–15 (AOR: 1.42, 95% CI: 1.03–2.37). Those with no formal education were about six times more likely to engage in risky sexual activity than those with a formal education (AOR: 5.73, 95% CI: 1.49–10.51). In addition, those street children who have lived on the street for more than 1 year were two times more likely to practice risky sexual practices (AOR: 2.14, 95% CI: 1.03–4.12). Those street children who drink alcohol and other substances were 24% more likely to engage in risky sexual activity as compared to those who did not drink (AOR: 1.24, 95% CI: 1.03–2.07). Furthermore, those street children with a daily average income of more than 21 Birr were about 32% less likely to engage in risky sexual practices as compared to those with a daily average income of less than 20 Birr (AOR: 0.68, 95% CI: 0.12–0.98) (Table 5).

**Table 5:** Bivariate and multivariate analyses of factors associated with risky sexual practice among street children in Wonago town, September 2021 (*N* = 214).

Variables	Risky sexual practice		COR (95%CI)	AOR (95%CI)
	Yes	No		
*Age*				
10–15	42	37	1	1
16–18	51	84	0.53 (0.3, 0.94)	1.42 (1.03, 2.37) *
*Sex*				
Male	88	115	0.92 (0.27, 3.11)	-
Female	5	6	1	
*Place of birth*				
Urban	47	62	0.97 (0.57, 1.67)	-
Rural	46	59	1	
*Educational level*				
No Formal Education	52	35	3.12 (1.77, 5.5)	5.73 (1.49, 10.51) *
Formal education	41	86	1	1
*Types of street children*				
On the street	72	71 2.41(1.32, 4.43) 1.05 (0.05, 3.05)		
Off the street	21	50 1 1		
*Substance use*				
User	69	63	2.65 (1.47, 4.75)	1.24 (1.03, 2.07) *
Non-user	24	58	1	1
*Duration on the street*				
≤1 yr.	16	37	1	1
>1 yrs.	77	84	2.12 (1.09, 4.11)	2.14 (1.03, 4.12) *
*Average daily income*				
<20	65	76	1	1
> = 21	28	45	0.73 (0.41, 1.29)	0.68 (0.12, 0.98) *
*Support from any organization*				
Yes	19	23	1.09 (0.56, 2.16)	1.41 (0.76–2.15)
No	74	98	1	

Hint: *p value < 0.05.

## 4. Discussion

In the present study, the magnitude of risky sexual practices was 43.46%. This implies that many street children are involved in risky sexual practices and are vulnerable to HIV and other sexually transmitted infections. The result of this study was much lower than the studies done in Brazil (61.9%) (19), Kenya (55%) (20), Gondar (61.7–62.4%) (21, 22), and Dilla Town, Ethiopia (53.9%) (23). The discrepancy with the Brazil study might be due to the socio-demographic variation such as sex, educational status, income, and differences in years living on the street between the two study areas (in the study of Brazil, half of the participants had been living on the street for at least 4 years, and in this study area only 17.6% lived for more than 4 years), since the variable of duration on the street is directly associated with risky sexual practice (19). The difference between this study and other Ethiopian studies could be attributed to the study participants’ age groups, as this study included younger age groups of 10–18 years old as opposed to older age groups of 15–24 years. In addition, the other possible reason for the difference might be the small sample size used in this study.

This study’s finding was also slightly higher than the study that was conducted in southern Ethiopia (31.6%) (15). The possible explanation for this difference might be the difference in study variables used to measure the risk of sexual practice. For example, the study in southern Ethiopia did not consider age at first sexual intercourse when measuring risky sexual practices. In addition, this study was done in the specific age group of 10–18, which is a highly risky group for risky sexual practices, unlike the study of southern Ethiopia, which was done on all street dwellers (15).

According to the findings of this study, 43.5% of participants had more than one sexual partner. This shows that more than half of street children have had sexual relationships with multiple partners. So, they are vulnerable to many sexually transmitted diseases due to the fact that sexual contact with multiple partners is one of the risk factors for sexually transmitted infections. This study’s finding is lower than the study done on Gondar, which is 91.7%. This might be due to the high prevalence of sexual practice (61.7%) in Gondar and the age group variation between the two studies. About half (51.4%) of the respondents in the Gondar study were in the age group of 15–19 years, unlike in this study, which was 10–15 (63.1%) (21). In addition, the finding of this study is lower than that of the study conducted in Kenya, Nairobi (23.3%) (18), which might be due to the fact that the study in Nairobi assessed participants’ number of partners with whom they had sex in the 3-month period prior to the study, whereas this study assessed the lifetime sexual partners of respondents.

From the findings of this study, 57% of sexually active street children used condoms during sexual intercourse, but 56% of them used inconsistently. This indicated that many sexually active street children never or inconsistently used condoms during sexual intercourse, and those individuals are at risk for sexually transmitted infections. This study result is comparable with the finding of a study conducted in Nairobi, Kenya, which showed 60.7% of respondents use condoms, and 58.5% of them use them inconsistently. But this finding is lower than the study done on Gondar, in which condom utilization was 74 and 80.5% of them were used in an irregular pattern. This discrepancy might be due to the study period differences between the two studies; the Gondar study was conducted in 2013 (21). Similarly, about 47.3% of street children have not used condoms due to a lack of access. This suggests that accessibility and availability of health services in health facilities, such as condoms, are very essential for people to protect themselves from HIV and other sexually transmitted diseases. This finding is supported by a study conducted in Addis Ababa and the southern part of Ethiopia, in which lack of access to sexual and reproductive health services was the most common reason for not utilizing those services (15, 17).

The current study revealed that children’s age, educational status, daily income, duration on the street, and status of substance use were significantly associated with risky sexual practices. Accordingly, being in the age group of 16–18 was 42% more likely to engage in risky sexual activity as compared to those in the age group of 10–14. This indicates that children near the age of 18 are more likely to engage in risky sexual practices than the lower age groups. This finding is supported by the fact that individuals in the indicated age groups are more sexually active. A comparable finding was reported in studies from Sri Lanka (27), Gondar (22) and Southern Ethiopia (15, 23) and other study (13). It could be because older street children, who have been living on the streets for longer, take drugs more frequently, increasing their intention to engage in risky sexual activity. Regarding educational status, in this study, children with no formal education were about six times more likely to engage in risky sexual activity than those with a formal education. This data demonstrates the significance of education in reducing the prevalence of risky sexual practices. This finding is supported by a study conducted in the southern part of Ethiopia (23), in which a negative association was observed between educational level and the magnitude of risky sexual practices. This may be because young people with no formal education are more likely to engage in high-risk sexual behaviors due to poor risk perception and a lack of sustained education on the issue. Furthermore, our study is in line with an adult study in Addis Ababa (28).

In addition, street children who have lived on the street for more than 1 year were two times more likely to practice risky sexual practices. This is supported by a study done in Gondar, Ethiopia (21) in which a longer duration of stay on the street was one of the predictors for risky sexual practice. It might be due to the fact that the more people who stay on the street, the more exposed they are to street subcultures such as smoking, drugs, alcohol, substance abuse, and sexual activities (17). Street children who drank alcohol and other substances were 24% more likely to engage in risky sexual activity as compared to those who did not drink. This result is supported by a study conducted in Sri Lanka (27), Dilla (23), and Gondar, Ethiopia (22). Substance abuse, such as alcohol use, may contribute to the development and practice of risky sexual behavior. Moreover, alcohol may reduce judgment and behavior *via* physiological or socially learned mechanisms.

Furthermore, in the present study, street children with a daily average income of more than 21 birr were about 32% less likely to engage in risky sexual practices as compared to those with a daily average income of less than 20 birr. This suggests that children with very low daily income are more likely to engage in risky sexual practices, which could be due to their need to engage in sexual activities to survive in life (known as “survival sex”). It clearly indicates that in order for street children to avoid engaging in sex for money, their daily income is essential. The finding is supported by a study conducted in southern Ethiopia (15).

### 4.1. Strengths and limitations of the study

As a strength, the study has focused on marginalized and neglected groups of people who are highly vulnerable to HIV and other STDs, where adequate information and studies are lacking. This might certainly fill some of the knowledge gaps and serve as baseline information for future studies. It is important to consider the following limitations when interpreting the results of this study: First, we used a face-to-face interview, which may not be practical to study sexuality in a conservative community. This might introduce social desirability bias, though the survey was done anonymously by arranging a same sex interviewer. Additionally, the study topic by itself assesses personal and sensitive issues related to sexuality, which might have caused underreporting of some behaviors. Second, recall bias cannot be ruled out, as the majority of children had dropped out of school at the primary level, leading to an overall lower education level in the group. Finally, due to the nature of the study design, it may not show cause and effect, so it should be repeated with a different study design.

## 5. Conclusion and recommendation

Despite the efforts taken to address sexual and reproductive health issues among street children, research results showed that risky sexual practices among street children were prevalent in Wonago Town, Southern Ethiopia. Risky sexual practice is more common among “on the street children,” and living on the street by itself is a contributing factor to child sexual abuse. Nearly half of the children in Wonago town still do not have access to basic sexual and reproductive health services such as condoms. Children’s age, educational status, daily income, duration on the street, and substance use were the factors contributing to risky sexual practices. As a result, conducting information, education, and communication campaigns—or behavioral change communication campaigns—about STDs, HIV/AIDS, substance use, and other risky sexual practices through peer education schemes, outreach programs, helplines or hotlines, and collaboration with HIV/AIDS organizations are important recommendations to reduce the occurrence of risky sexual practices among street children. Providing capacity-building training for health care providers may decrease the risk of risky sexual practices. In addition, reducing children’s duration of stay on the street by creating income-generating activities, which subsequently improve children’s daily income, and increasing school attendance are other very important recommendations to reduce the magnitude of risky sexual practice. Furthermore, improving access to sexual and reproductive health services and making them available free of charge, if possible, at all health facilities might decrease the risk of risky sexual practices among street children.

## Data availability statement

The raw data supporting the conclusions of this article will be made available by the authors, without undue reservation.

## Ethics statement

The studies involving human participants were reviewed and approved by Institutional Review Board (IRB) of Dilla University College of Medicine and Health Sciences. Written informed consent to participate in this study was provided by the participants’ legal guardian/next of kin.

## Author contributions

GAZ and WA drafted the design, prepared the data collection tool, and wrote the paper. GAZ, BGD, and WA performed the data analyses. GAZ, TM, and WA reviewed and revised the paper. All authors have read and approved the final manuscript.

## Conflict of interest

The authors declare that the research was conducted in the absence of any commercial or financial relationships that could be construed as a potential conflict of interest.

## Publisher’s note

All claims expressed in this article are solely those of the authors and do not necessarily represent those of their affiliated organizations, or those of the publisher, the editors and the reviewers. Any product that may be evaluated in this article, or claim that may be made by its manufacturer, is not guaranteed or endorsed by the publisher.
